# Retraction Note: MicroRNA-221-3p, a TWIST2 target, promotes cervical cancer metastasis by directly targeting THBS2

**DOI:** 10.1038/s41419-022-05469-2

**Published:** 2022-12-02

**Authors:** Wen-Fei Wei, Chen-Fei Zhou, Xiang-Guang Wu, Li-Na He, Lan-Fang Wu, Xiao-Jing Chen, Rui-Ming Yan, Mei Zhong, Yan-Hong Yu, Li Liang, Wei Wang

**Affiliations:** 1 Department of Obstetrics and Gynecology, Nanfang Hospital/First School of Clinical Medicine, Southern Medical University, Guangzhou, Guangdong Province People’s Republic of China; 2 Department of Obstetrics and Gynecology, Third Affiliated Hospital, Southern Medical University, Guangzhou, Guangdong Province People’s Republic of China; 3 Department of pathology, Nanfang Hospital/First School of Clinical Medicine, Southern Medical University, Guangzhou, Guangdong Province People’s Republic of China; 4 Department of Obstetrics and Gynecology, First Affiliated Hospital, Guangzhou Medical University, Guangzhou, Guangdong Province People’s Republic of China

Retraction to: *Cell Death and Disease* 10.1038/s41419-017-0077-5, published online 14 December 2017

The Editors-in-Chief have retracted this article because of significant concerns regarding Figure 2C, Supplementary Figures 5 and 6 presented in this work, which question the integrity of the data. Therefore, the Editors-in-Chief, no longer have confidence in the integrity of the data in this article. Authors Wen-Fei Wei, Xiaojing Chen, Lina He, Xiangguang Wu and Wei Wang have not explicitly stated whether they agree to this retraction notice. The editors were not able to obtain a current email address for Mei Zhong, Yan-Hong Yu and Li Liang. Chen-Fei Zhou, Lan-Fang Wu, and Rui-Ming Yan have not responded to any correspondence from the editor/publisher about this retraction.

